# Preparation of “Ion-Imprinting” Difunctional Magnetic Fluorescent Nanohybrid and Its Application to Detect Cadmium Ions

**DOI:** 10.3390/s20040995

**Published:** 2020-02-13

**Authors:** Lina Chen, Yue Lu, Minshu Qin, Fa Liu, Liang Huang, Jing Wang, Hui Xu, Na Li, Guobao Huang, Zhihui Luo, Baodong Zheng

**Affiliations:** 1 Guangxi Key Laboratory of Agricultural Resources Chemistry and Biotechnology, Yulin 537000, China; lnchen2018@163.com (L.C.); qms5566@163.com (M.Q.); fa.l@163.com (F.L.); ln19860622@126.com (N.L.); lzjx0915@163.com (G.H.); 2 Colleges and Universities Key Laboratory for Efficient Use of Agricultural Resources in the Southeast of Guangxi, Yulin 537000, China; 3 College of Chemistry and Food Science, Yulin Normal University, Yulin 537000, China; 4 Engineering Research Center of Marine Living Resources Integrated Processing and Safety Risk Assessment, College of Food Science, Fujian Agriculture and Forestry University, Fuzhou 350002, China; luyue1106@126.com (Y.L.); xhuifst@163.com (H.X.); 5 College of Chemical Engineering, Zhejiang University of Technology, Hangzhou 310014, China; lhuang@zjut.edu.cn (L.H.); lovejllmy@163.com (J.W.)

**Keywords:** bifunctional, magnetic, fluorescent, Cd^2+^, detection, ion-imprinting

## Abstract

In this work, we have fabricated a novel difunctional magnetic fluorescent nanohybrid (DMFN) for the determination of cadmium ions (Cd^2+^) in water samples, where the “off-on” model and “ion-imprinting” technique were incorporated simultaneously. The DMFN were composed of CdTe/CdS core-shell quantum dots (QD) covalently linked onto the surface of polystyrene magnetic microspheres (PMM) and characterized using ultraviolet-visible spectroscopy (UV-Vis), fluorescence spectroscopy, and transmission electron microscopy (TEM). Based on the favorable magnetic and fluorescent properties of the DMFN, the chemical etching of ethylene diamine tetraacetic acid (EDTA) at the surface produced specific Cd^2+^ recognition sites and quenched the red fluorescence of outer CdTe/CdS QD. Under optimal determination conditions, such as EDTA concentration, pH, and interfering ions, the working curve of determining Cd^2+^ was obtained; the equation was obtained Y = 34,759X + 254,894 (*R* = 0.9863) with a line range 0.05–8 μM, and the detection limit was 0.01 μM. Results showed that synthesized magnetic fluorescent microspheres had high sensitivity, selectivity, and reusability in detection. Moreover, they have significant potential value in fields such as biomedicine, analytical chemistry, ion detection, and fluorescence labeling.

## 1. Introduction

As a heavy metal ion with strong toxicity, Cd^2+^ has a long half-life, uneasy degradation in vivo, and readily builds up in human tissues. It can cause acute or chronic toxicity through the food chain and gives rise to symptoms such as degrading renal function, osteoporosis, and carcinogenesis [1,2]. Animal experiments have verified that cadmium will affect immunological functions of cells, manifested by the quantity of lymphocytes, transforming function, cytokines, and subpopulation change [3,4,5]. Therefore, in order to protect the environment and human health, it is important to establish a sensitive and highly efficient cadmium detection method.

Currently, methods to detect Cd^2+^ include atomic absorption spectrometry (AAS) [6], atomic fluorescence spectrophotometry (AFS) [7], inductively coupled plasma mass spectrometry (ICP-MS) [8], voltammetry [9,10,11], and the fluorescence sensor method [12,13,14]. To solve sample pretreatment, some research groups have been developing the high detection of Cd^2+^ combining magnetic separation [15,16,17,18]. Especially, the advantages of the fluorescence sensor methods are speed, visuality, and high sensitivity. Up to now, the multifunctional fluorescent probe has attracted attention in current studies due to its speed, high sensitivity, and multi-modal detection, and it has been widely applied in fields such as biomedicine and analytical chemistry.

Compared with other organic fluorescent probes, quantum dots (QD) have been applied extensively to fluorescence detection because of their unique optical characteristics: high fluorescent quantum yield, size-adjustable fluorescence-emission peak, and multiple fluorescence colors [19,20]. Furthermore, Cd-based QD were considered for producing a surface Cd^2+^-imprinted material using EDTA as chemical etching and applied to detect Cd^2+^ [12,21]. Magnetic nanoparticles are widely studied for their applications in biosensor, biology, and medicine due to excellent biocompatibility, outstanding magnetic imaging, superior separation [22,23], especially for small molecules detection, and pathogen and nonpathogen bacteria separation in a complex sample. Thus, the synthesis of bifunctional magnetic-fluorescent particles has become a hot topic for the quick detection and multiple imaging technologies.

**Scheme 1:**
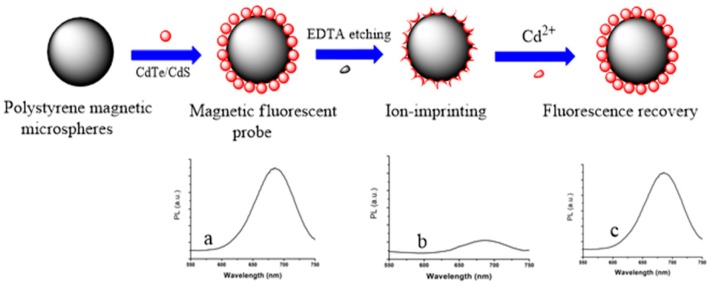
Schematic illustration for synthesis of difunctional magnetic fluorescent nanohybrid (DMFN) and the sensing mechanism for fluorescence off-on detection of Cd^2+^.

In this study, based on the superior characteristics of multifunctional fluorescent probes, we have constructed an “ion-imprinted” difunctional magnetic fluorescent nanohybrid as a fluorescent probe for off-on sensing of Cd^2+^. As illustrated in Scheme 1, the DMFN were obtained through CdTe/CdS QD covalently linked onto the surface of amino polystyrene magnetic microspheres (PMM). The system of detecting Cd^2+^ was established according to the strong complexing action between EDTA and Cd^2+^ to produce the Cd^2+^-imprinted cavities, by optimizing conditions such as EDTA concentration, pH, and interfering ions; the optimal determination conditions were achieved and successfully applied to the detection of cadmium ions in water. Results showed that the synthesized magnetic fluorescent microsphere probe had high sensitivity, selectivity, and consistency of detection.

## 2. Experimental

### 2.1. Material and Methods

All the starting materials were used without further purification. Cadmium chloride, trisodium citrate, sodium tellurate, and sodium borohydride were obtained from Sinopharm Chemical Regent Co. Ltd.. N-Ethyl-N’-(3-dimethylaminopropyl)carbodiimide hydrochloride (EDC) was purchased from Aladdin Chemicals Co. Ltd.. Amino-polystyrene magnetic microspheres (PMM) were obtained from Tianjin BaseLine Chrom.Tech. Research Centre. For all aqueous solutions, high-purity ultrapure water from a Millipore (18.2 MΩ·cm) system was used throughout the experiments.

### 2.2. Measurements

The high-resolution transmission electron microscopy (HRTEM) of DMFN was obtained using a JEM-2010FEF microscope (JEOL, Tokyo, Japan). Ultraviolet-visible (UV-Vis) absorption spectra were acquired on a Cary 5000 UV-Vis-NIR spectrophotometer (Agilent Technologies, Santa Clara, CA, USA) coupled with a 1.00 cm quartz cell. All photoluminescence (PL) spectra were performed with FluoroMax-4 (HORIBA, Tokyo, Japan).

#### 2.2.1. Synthesis of CdTe/CdS Quantum Dots

The one-pot method was used for the hydro-thermal synthesis of CdTe/CdS QD [24]. In a typical synthesis, 0.1142 g cadmium chloride was dissolved in 250 mL of ultrapure water, and then 0.2690 g of trisodium citrate and 50 μL MPA were successively added under magnetic stirring for 15 min, followed by adjusting the pH to 11.2 by dropwise addition of 1.0 mol·L^−1^ sodium hydroxide solution, and then 0.0222 g sodium tellurate and 0.1 g sodium borohydride were added under magnetic stirring, and refluxed in a 90 °C oil bath. After a four-hour reaction time, red emitting quantum dots were produced. In order to improve the chemical stability of the CdTe/CdS QD, the CdTe/CdS QD solution was refined by cooling the synthesized quantum solution to room temperature. The products were purified three times by centrifugation at 5000 rpm, and the resultant precipitates were re-dissolved in ultrapure water for further use.

#### 2.2.2. Synthesis of DMFN

Two milliliters of EDC (2 mg/mL) and 2 mL CdTe/CdS QD were mixed, and the pH was adjusted to 6.5 using 0.01 mol/L HCl, and then it was stirred for 30 min for follow-up usage. Fifteen milliliters of PMM (0.035 mg/mL) was added under magnetic stirring, followed by adjusting the pH to 8.5–9 by dropwise addition of 0.01 mol/L NaOH, and placed in 4 °C dark conditions for 12 h. After reaction, the mixture was centrifuged repeatedly, and quantum dots that did not react in the supernatant were discarded until fluorescence in the supernatant did not reduce any further. The products were purified three times by centrifugation at 4000 rpm, and then the DMFN were obtained.

#### 2.2.3. DMFN for Detecting Cd^2+^ in Water

All fluorescence spectra measurements were carried out under the same conditions at room temperature. A typical procedure for the detection of Cd^2+^ is described as follows: 300 μL of DMFN (0.2 mg·mL^−1^), 600 μL of Tris-HCl buffer (pH = 8.5), and 24 μL of EDTA (10 μM) were added to a 4 mL PE tube, and certain amounts of Cd^2+^ were sequentially added. The mixture was then diluted to 3 mL with ultrapure water and mixed thoroughly. Ten minutes later, the mixture was transferred to a 4 mL cuvette, and the fluorescence intensity of the solution was recorded under the excitation wavelength set at 380 nm with detect slit of 5 nm.

## 3. Results and Discussion

### 3.1. Characterization of DMFN

**Figure 1:**
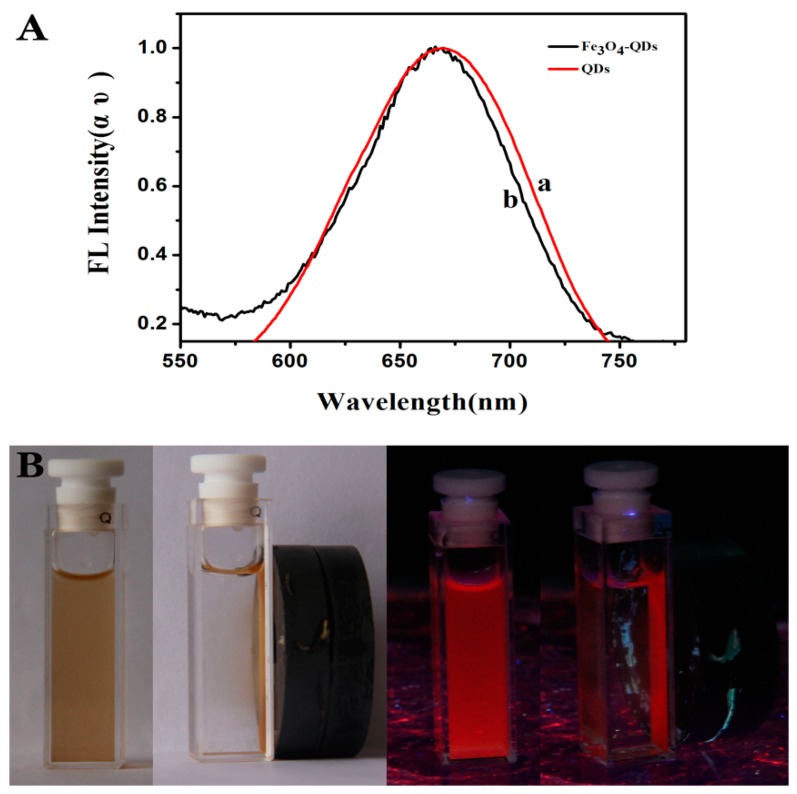
(**A**) the fluorescence spectra of CdTe/CdS QD (a) and the DMFN (b), and (**B**) the photograph of DMFN under white light (**left**) and UV light (**right**).

In order to verify whether the CdTe/CdS QD were successfully coupled on the magnetic microspheres, the DMFN were characterized using the fluorescent spectrometer and TEM. As presented in Figure 1, it can be seen from Figure 1A that the fluorescent spectrum of DMFN were similar to independent CdTe/CdS QD. It had a narrow and symmetrical fluorescence spectrum with the highest peaks at 680 nm when the exited wavelength was 380 nm. Furthermore, the DMFN could quickly be gathered when the magnet was near the side of the cuvette and showed strong red PL under the UV lamp (Figure 1B). It indicated that the CdTe/CdS QD were successfully adsorbed onto the magnetic microspheres and that the difunctional magnetic fluorescent probe was synthesized with obvious fluorescence intensity and magnetism.

**Figure 2:**
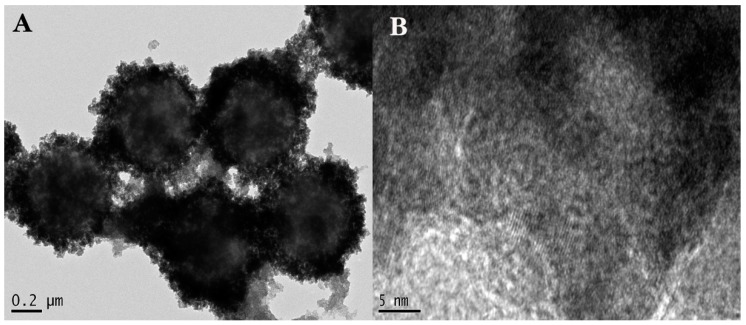
(**A**)Transmission electron microscopy (TEM) images of the DMFN; (**B**) High-resolution TEM images of the DMFN.

The morphology and size of the DMFN were characterized by HRTEM, and the results are shown in Figure 2. Numerous, dark “QD islands” along the edge of the PMM can be observed from Figure 2A. Furthermore, a tiny oblique line can be seen in Figure 2B, and this shows that there were obvious and uniform crystal lattices on the surface of the magnetic microspheres. The existence of well-resolved crystal lattices in HRTEM images further confirmed the excellent crystalline structures of the CdTe/CdS quantum dots. It is worth mentioning that the particle sizes of the CdTe/CdS quantum dots were about 4.5 nm. It also indicates that CdTe/CdS quantum dots were successfully adsorbed on the magnetic microspheres.

**Figure 3:**
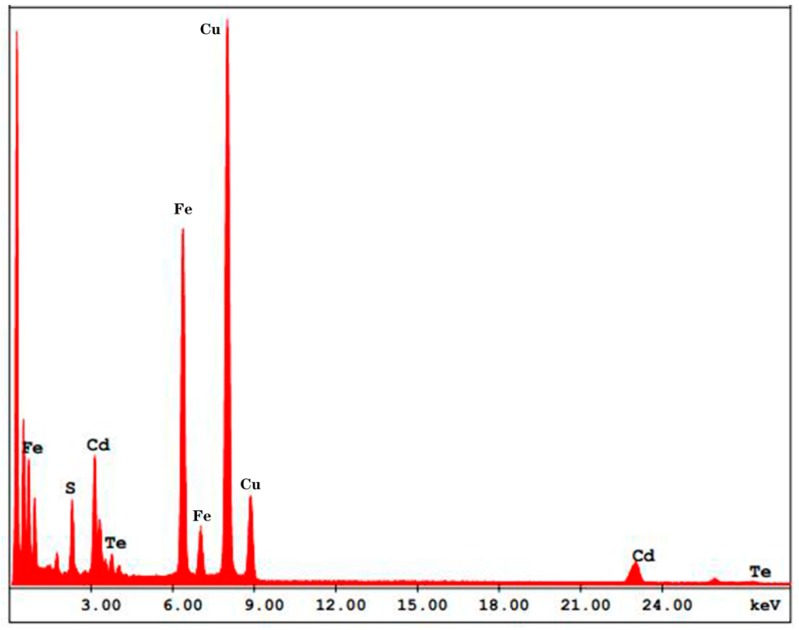
The energy dispersive spectrum of DMFN.

In order to further verify the CdTe/CdS QD were successfully adsorbed on the magnetic microspheres, the energy dispersive spectrum (EDS) analysis of the DMFN was conducted (Figure 3). It can be seen in Figure 3 that O, Fe, S, Cd, and Te elements were present on the DMFN, and the peaks of Cu element came from the copper grid during the detecting. These results show that CdTe/CdS quantum dots were successfully adsorbed on the magnetic microspheres and that the bi-functional magnetic fluorescent probe was successfully synthesized.

### 3.2. Determination Mechanism of Cd^2+^

Based on the strong complexing action between EDTA and Cd^2+^ to produce the Cd^2+^-imprinted cavities in this experiment, imprinting of Cd^2+^ on the quantum surface on magnetic fluorescent microspheres could be realized, which created defects on the quantum surface and resulted in degraded fluorescence intensity, and the results are shown in curve b in Scheme 1. When additional cadmium ions were added in the system, they repaired the defects of the quantum dots on the microsphere surface automatically, so that the fluorescence was recovered. The results are shown by curve c in Scheme 1. Moreover, the fluorescence intensity was enhanced as Cd^2+^ concentration increased. A rapid, sensitive, and highly selective determination method of Cd^2+^ was established based on this off-on mechanism.

### 3.3. Influence of Differing pH on the DMFN

**Figure 4:**
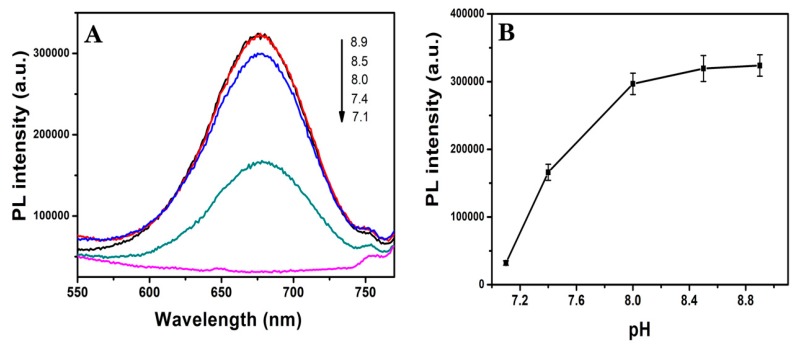
(**A**) fluorescence spectra of DM**FN** under different pH and (**B**) PL intensity of DMFN under different pH on the system.

In order to improve the experimental sensitivity, a series of Tris-HCl buffer solutions with different pH values were measured through fluorescence spectra. As shown in Figure 4, the fluorescence intensity of DMFN was increased in the range of pH values from 7.1 to 8.9 in the etching reaction (Figure 4B), and the DMFN exhibited a relatively high intensity at pH 8.5, so the optimum pH 8.5 was chosen to use in the experiment.

### 3.4. Influence of Different Concentrations of EDTA on the Detection System

**Figure 5:**
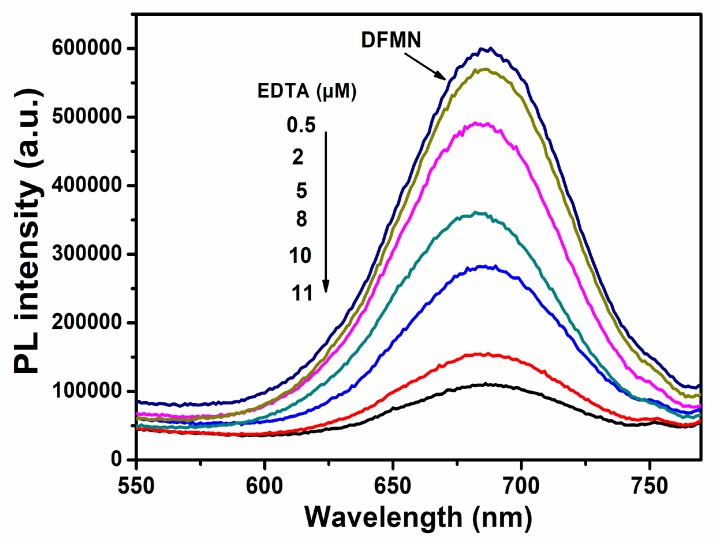
Fluorescence spectra of DMFN under different concentrations of EDTA.

To further optimize the experimental conditions and improve its sensitivity, different concentrations of EDTA were added for optimization. As shown in Figure 5, it demonstrated that the fluorescence intensity of DMFN was quenched when EDTA concentration increased due to its etching the outer CdTe/CdS QD on the surface of DMFN. More EDTA was added, more Cd^2+^ were replaced, and this produced more defects on quantum surface, so the fluorescence intensity of DMFN was quantitatively quenched. For a compromise between the quenching efficiency and recovery ability, the EDTA concentration of 10 μM was selected.

### 3.5. Influence of Different Metal Ions on the Detection System

**Figure 6:**
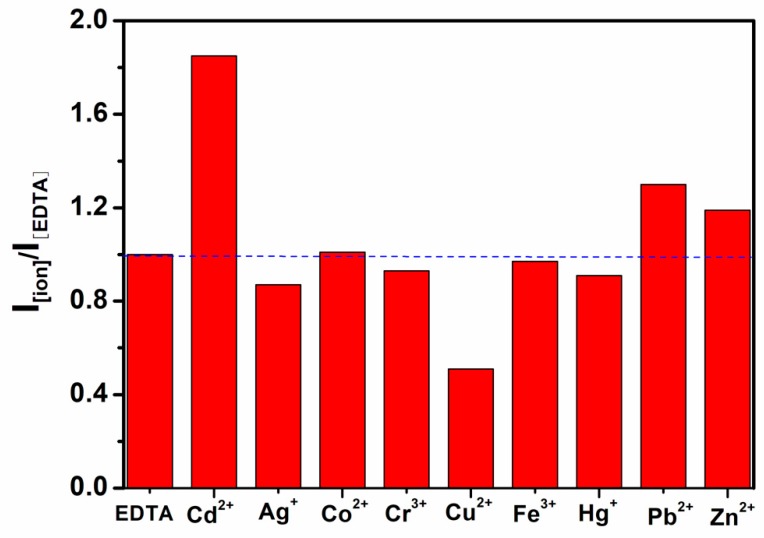
Influence of different metal ions on the system in the Tris-HCl buffer.

**Figure 7:**
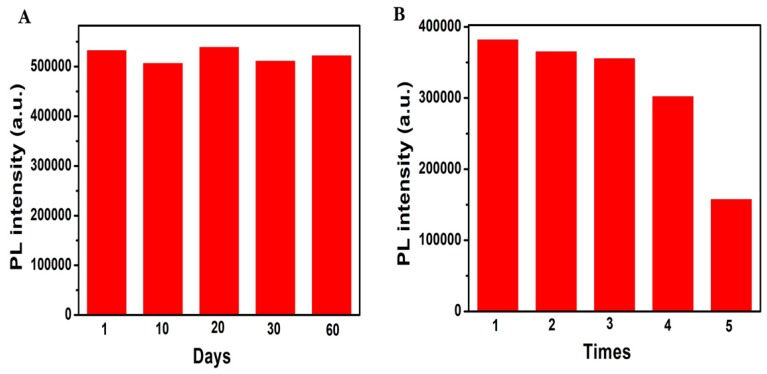
**(A**) PL intensity of DMFN under different days and (**B**) PL intensity of DMFN under different recyclability times on the system.

To examine the selectivity of the Cd^2+^-imprinted DMFN, Cd^2+^, Ag^+^, Co^2+^, Cr^3+^, Cu^2+^, Fe^3+^, Hg^2+^, Pb^2+^, and Zn^2+^ were used as interfering ions, and the verification experiment was carried out according to the experimental method. The results are shown in Figure 6. Under the same concentration, it can be seen that the PL intensity ratio of I_[ion]_/I_[EDTA]_ in most of the ions was lower, while the Cd^2+^ were higher in a remarkable intensity. This shows that these ions do not influence the veracity of this method, and Cd^2+^ significantly enhance the fluorescence, indicating that this method has favorable detection selectivity. Furthermore, the stability and recyclability properties of DMFN were tested. For evaluating the stability of DMFN, we measured PL intensity of DMFN using 300 μL of DMFN (0.2 mg·mL^−1^) under the excitation wavelength set at 380 nm with detect slit of 5 nm. As shown in Figure 7, the PL intensity of DMFN did not change much more in at least two months. It indicated that the DMFN have good stability. Furthermore, the DMFN can be recyclable three times with the concentration of Cd^2+^ (4 μM).

### 3.6. Determination of Cadmium Ions in Water

**Figure 8:**
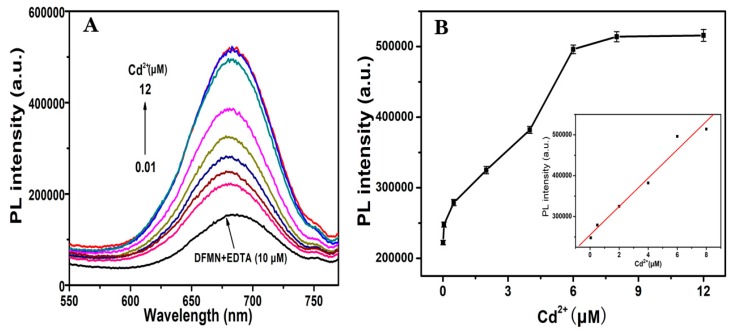
(**A**) fluorescence spectra of DMFN under different concentrations of Cd^2+^ and (**B**) plot of the photoluminescence (PL) intensity of the DMFN as a function of the Cd^2+^ concentration.

To test the feasibility of DMFN for detecting Cd^2+^, under optimal conditions, different concentrations of Cd^2+^ were added to the DMFN, and the change in fluorescence intensity was examined. As shown in Figure 8, as the concentration of Cd^2+^ increased, the fluorescence intensity of DMFN was gradually recovered. A good linear relationship was obtained, Y = 34,759X + 254,894 (*R* = 0.9863) with a line range 0.05–8 μM, and the detection limit was 0.01 μM.

Based on the better selectivity and sensitivity of the DMFN in the buffer solutions, we further applied it to the detection of actual water samples. The result is shown in Table 1. The standard recovery rate of Cd^2+^ was detected at room temperature, and the recovery was calculated to be between 80.5% and 89.3% by linear regression equation, which further proves the reliability of this method.

## 4. Conclusions

In summary, we have demonstrated the DMFN were assembled using magnetic microspheres and CdTe/CdS quantum dots as functional materials for the fluorescence detection of Cd^2+^ via off-on model with “ion-imprinting” techniques. It has been shown that CdTe/CdS quantum dots were successfully adsorbed on the magnetic microsphere surface through characterizations of visible absorption spectrum, fluorescence spectrum, and TEM. The DMFN relies on the chemical etching of Cd^2+^ by EDTA on the surface of outer CdS QD, and the red fluorescence of the DMFN is quenched due to the increased surface defects. A good linear relationship was obtained, Y = 34,759X + 254,894 (*R* = 0.9863) with a line range 0.05–8 μM, and the detection limit was 0.01 μM. The synthesized DMFN has significant potential application in ion detection, cell separation, and the detection and targeting of drugs, among other uses.

## Figures and Tables

**Table 1:** Determination of water samples using this method.

Place	Measured Value of the Sample (μM)	Added Standard Value (μM)	Measured Standard Value (μM)	Recovery	Average Recovery
Estuary of River of Hepu	0	2	1.61	80.5	85.95
	0	6	5.25	87.5	
Nanliu River of Yulin	0	2	1.73	86.5	
	0	6	5.36	89.3	
